# Analysis of the role of dihydromyricetin derived from vine tea (*Ampelopsis grossedentata*) on multiple myeloma by activating STAT1/RIG-I axis

**DOI:** 10.32604/or.2024.043423

**Published:** 2024-07-17

**Authors:** WEI JIANG, MEI ZHOU

**Affiliations:** 1 Department of Hematology, Shaoxing Shangyu People’s Hospital, Shaoxing, 312000, China; 2 Department of Hematology, Zhuji People’s Hospital, Shaoxing, 311800, China

**Keywords:** Dihydromyricetin, Multiple myeloma, Epithelial-mesenchymal transition, Tumor growth

## Abstract

Multiple myeloma (MM) is a plasma cell malignancy and remains incurable as it lacks effective curative approaches; thus, novel therapeutic strategies are desperately needed. The study aimed to explore the therapeutic role of dihydromyricetin (DHM) in MM and explore its mechanisms. Human MM and normal plasma samples, human MM cell lines, and normal plasma cells were used for *in vitro* experiments. Cell counting kit-8 (CCK-8), flow cytometry, and trans-well assays were performed for the assessment of cell viability, apoptosis, migration, and invasion, respectively. Quantitative real-time polymerase chain reaction (qRT-PCR) was employed to assess the mRNA expression of signal transducer and activator of transcription 1 (STAT1) and retinoic acid-inducible gene I (RIG-I). Western blotting was employed to assess E-cadherin, N-cadherin, signal transducer, STAT1, p-STAT1, and RIG-I protein expression. A tumor xenograft model was used for *in vivo* experiments. Here, dihydromyricetin (DHM) dose-dependently restrained viability, apoptosis, migration, and invasion, and facilitated apoptosis of U266 cells. After DHM treatment, the E-cadherin level was increased and the N-cadherin level was decreased in U266 and RPMI-8226 cells, suggesting the inhibitory effects of DHM on epithelial-mesenchymal transition (EMT) in MM. Besides, the levels of p-STAT1/STAT1 and RIG-I were down-regulated in MM. However, the STAT1 inhibitor fludarabine undid the suppressive effect of DMH on the malignant characteristics of U266 cells. Also, DHM inhibited MM tumor growth and EMT, and activated STAT1/RIG-I pathway *in vivo*. Collectively, this study first revealed that DHM can restrain EMT and tumor growth in MM by activating STAT1/RIG-I signaling, which provides a novel drug for the treatment of MM.

## Introduction

Multiple myeloma (MM) is a severe plasma cell disorder, with rising prevalence in developed countries, like the US and Australia [1,2]. MM is the second most frequent hematologic malignancy featured by aberrant bone marrow clonal plasma cell accumulation, diagnosis of plasmacytoma, monoclonal protein in serum or urine, and end-organ injury signs [2–4]. MM implicates a series of clinical variants, from benign monoclonal gammopathy of uncertain significance (MGUS) and smoldering/indolent MM to more destructive, disseminated MM modalities and plasma cell leukemia [5]. To date, some drugs such as bortezomib, carfilzomib, daratumumab, and dexamethasone have been applied for MM treatment [6–8]. Despite these improvements, MM remains incurable; further investigation into the pathogenesis of MM and novel drugs is required.

Dihydromyricetin (DHM), also named ampelopsin, is a flavonoid extracted from the leaves and stems of the edible plant *Ampelopsis grossedentata* that is widely applied in traditional Chinese medicine (TCM) [9,10]. DHM has exhibited a broad range of pharmacological properties, like anti-inflammatory, anti-oxidative, anti-diabetic, and anti-tumor effects [11–14]. DHM has attracted wide attention due to its potent effects on inhibiting lung cancer, colon cancer, ovarian cancer, breast cancer, and cholangiocarcinoma [15–19]. Additionally, DHM has been used for multiple biological activities, involving cell proliferation, migration, invasion, and apoptosis [20]. Previous research showed that DHM could ameliorate hepatocellular carcinoma by suppressing hepatoma cell migration and invasion [21]. Nonetheless, whether DHM also exerts anti-MM effects remains unknown 4. Therefore, this study explored the effects of DHM on MM and related mechanisms.

Epithelial-mesenchymal transition (EMT) is an organized, polygenic biological process, during which the epithelial cell undergoes depolarization and acquires mesenchymal traits. EMT is a pivotal factor in cancer cell invasion, migration, metastasis, and chemoresistance [22,23]. EMT plays a vital part in cancer development and progression. Evidence suggests that modulating EMT-related signal transduction can mitigate cholangiocarcinoma [24], and regulating the expression of EMT markers can inhibit colorectal cancer [25]. Evidence shows that DHM exerts suppressive impacts on cancer through suppression of EMT [26–28]. For instance, DHM inhibits EMT by targeting the miR-455-3p axis in cholangiocarcinoma [19] and TNF-α/NF-κB axis in breast cancer [28]. Besides, retinoic acid-inducible gene I (RIG-I) is a multifunctional protein that can interact with signal transducer and activator of transcription 1 (STAT1), which acts as a switch in anti-tumor activity [29–31]. Nevertheless, the implication of STAT1/RIG-I axis in EMT and tumor growth in MM is still unclear.

In this research, the inhibitory effects of DHM on EMT and tumor growth in human MM were explored both *in vitro* and *in vivo*. Otherwise, DHM was found to exert inhibitory effects on EMT and tumor growth by activating the STAT1/RIG-I pathway in MM. Overall, this work first investigated the therapeutic effects of DHM on MM and the role of STAT1/RIG-I pathway in EMT in MM. This is expected to provide a scientific basis for the formulation of innovative therapeutic strategies for MM and fresh perspectives into the molecular mechanisms underlying MM development.

## Materials and Methods

### Patients and clinical samples

The tumor plasma samples were obtained from 15 patients with MM in Zhuji People’s Hospital (9 patients) and Shaoxing Shangyu People’s Hospital (6 patients) from January 14, 2022 to February 23, 2022. Besides, the normal plasma samples were collected from 15 healthy volunteers and were registered as normal controls in Zhuji People’s Hospital (9 volunteers) and Shaoxing Shangyu People’s Hospital (6 volunteers) from January 14, 2022 to February 23, 2022. The acquired samples were kept at −70°C. This study was approved by the Ethics Committee of the Zhuji People’s Hospital (Ethics Code: 062301) on June 23, 2022 and Shaoxing Shangyu People’s Hospital (Ethics Code: 20220223SYYY) on June 24, 2022. All patients signed informed consent. This research was conducted ethically in accordance with the World Medical Association Declaration of Helsinki.

### Cell culture and treatments

Human MM cell lines (U266, RPMI-8226, and NCI-H929) and normal plasma cells (nPCs) (American Type Culture Collection, Manassas, VA, USA) were cultivated in Roswell Park Memorial Institute (RPMI)-1640 Medium (HyClone, Logan, UT, USA) containing 10% fetal bovine serum (FBS; #S9030, Solarbio, Beijing, China) at 37°C with 5% CO_2_. DHM (HPLC ≥ 98%; #S24435, Shanghai Yuanye, Shanghai, China) was dissolved in dimethyl sulfoxide (DMSO; #HY-Y0320, MedChemExpress, Shanghai, China), and maintained at −20°C. The stock solutions were diluted to the different concentrations for later use. The ultimate DMSO concentration consistently remained below 0.2% (v/v). Different doses (50, 100, and 150 μM) of DHM were administered to U266 cells for 24 h. To further ascertain the underlying mechanism, phosphate buffer saline (PBS; #C0221A, Beyotime, Shanghai, China) or fludarabine (a STAT1 inhibitor; #M2028, AbMole, Shanghai, China), combined with 150 μM of DHM, was treated to U266 and RPMI-8226 cells. Cells without any treatments served as the control cells.

### Animal model and treatments in vivo

Twelve male BALB/c nude mice (6 weeks, 20 ± 2 g) used in this study were supplied by Shanghai SLAC Laboratory Animal (Shanghai, China). The BALB/c nude mice were randomly assigned to control and DHM groups (n = 6). All the mice were bred under the following conditions: a 12-h light/dark cycle; temperature, 25°C; and available food and water before the experiments.

U266 cells (5 × 10^6^ cells/mouse) were subcutaneously administered to the mice to establish a xenograft model according to previous studies [16,32]. The tumor volume (computed as length × width^2^/2) was monitored every 4 days following injection. Once the mean volume of the tumor came up to 100 mm^3^, the xenograft mice were intragastrically administered 100 mg/kg DHM (dissolved in normal saline) every day. Meanwhile, the control mice were administrated with an intragastric injection of the same amount of normal saline. Then, the body weights of mice, tumor weights, and volumes were monitored, followed by functional assays. After 21 days of administration, all the mice were euthanized by CO_2_ asphyxiation, away from other animals. The execution process was in accordance with previous research [33]. All animal experimental procedures were approved by the Animal Ethics Committee of Zhuji People’s Hospital (Ethics Code: 062301) and Shaoxing Shangyu People’s Hospital (Ethics Code: 20220223SYYY).

### Cell counting kit-8 (CCK-8) assay

The CCK-8 kit (#C0037, Beyotime, Shanghai, China) was applied to detect cell viability of the MM cell line, U266. U266 cells (2 × 10^4^ cells/mL) were seeded into 96-well platesfollowed by exposure to designated agents for 24, 48, and 72 h at 37°C with 5% CO_2_. Subsequently, CCK-8 solution (10 µL) was introduced to each well, and the optical density (OD) was assessed at 450 nm under a microplate reader (Hiwell Diatek, Wuxi, China) follwing 2 h of incubation.

### Flow cytometry

Flow cytometry was employed for the assessment of cell apoptosis via Apoptosis Detection Kit (#C1062S, Beyotime, Shanghai, China). The U266 cells with 48-h post-treatment were were subjected to three washes with PBS and then suspended in 300 μL binding buffer. Thereafter, staining of the cells was carried out by adding Annexin V-Fluorescein isothiocyanate (FITC; 5 μL) and propidium iodide (PI; 5 μL) Detection Kit (Beyotime, Shanghai, China) for 15 min in darkness. Finally, cell apoptosis was assessed on a flow cytometer (CytoFLEX S, Beckman, Miami, FL, USA) using Cell Quest software (BD Biosciences, Franklin Lakes, NJ, USA).

### Trans-well assays

Trans-well chambers were utilize to evaluate cell migration and invasion. Cells with 48-h post-treatment were adjusted to 1 × 10^5^/mL, and 200 µL cells were introduced into the upper chamber, which had been pre-coated with Matrigel for the trans-well invasion assay. The lower chamber was filled with RPMI-1640 Medium containing 10% FBS. Following a 24-h incubation period, the cells in the lower chamber were rinsed with PBS, fixed with methanol for 30 min, and subsequently stained with 0.1% crystal violet for 20 min. Finally, the cells were enumerated and captured by a microscope (DMi3000 B, Leica, Wetzlar, Germany).

### Quantitative real-time polymerase chain reaction (qRT-PCR) assay

Isolation of total RNA from MM tumor and normal plasma samples or cells (nPCs, U266, RPMI-8226, and NCI-H929) was realized via TRIzol reagent (#15596018, Invitrogen, Carlsbad, CA, USA) following the supplier’s protocols. Subsequently, the isolated RNA was reverse-transcribed into cDNA using the PrimeScript RT-PCR Kit (TaKaRa, Beijing, China). The Mx3000P Real-Time PCR System (Stratagene, CA, USA) was utilized in the experimental procedures, with the following conditions: 95°C, 3 min; 95°C, 12 s (40 cycles); 62°C, 40 s. Gene expression levels were determined using the 2^−ΔΔCt^ method, with GAPDH serving as the internal reference. Below were the primers used: STAT1 forward, 5′-TCA GGC TCA GTC GGG GAA TA-3′ and reverse, 5′-ATC ACT TTT GTG TGC GTG CC-3′; RIG-I forward, 5′-CTG GTT CCG TGG CTT TTT GG-3′ and reverse, 5′-AGC AGG CAA AGC AAG CTC TA-3′; GAPDH forward, 5′-TGT GGG CAT CAA TGG ATT TGG-3′ and reverse, 5′-ACA CCA TGT ATT CCG GGT CAA T-3.

### Western blotting

Total protein was separated using the radioimmunoprecipitation assay (RIPA) lysate (#P0013B, Beyotime, Shanghai, China) and quantified using a bicinchoninic acid (BCA) kit (#P0010S, Beyotime, Shanghai, China). Thereafter, the obtained proteins were transferred to several polyvinylidene difluoride (PVDF) membranes (#FFP24, Beyotime, Shanghai, China), which were blocked by 5% nonfat milk (#P0216, Beyotime, Shanghai, China) for 1 h. Then, the membranes were exposed to the primary antibodies and incubated at 4°C overnight, followed by three washes with Tris-buffered saline with Tween-20 (TBST; #ST677, Beyotime, Shanghai, China) for 10 min. Below were the primary antibodies employed: anti-E-cadherin (1:1,000; #AF0131, Affinity, San Antonio, TX, USA), anti-N-cadherin (1:5,000, #ab76011, Abcam, Cambridge, UK), anti-STAT1 (1:1,000; #AF6300, Affinity, San Antonio, TX, USA), anti-p-STAT1 (#AF3300, Affinity, San Antonio, TX, USA), and anti-RIG-I (1:1,000; #4200S, Cell Signaling Technology, Danvers, MA, USA), and anti-GAPHD (1:10,000; #ab245355, Abcam, Cambridge, UK). Next, the membranes were subjected to 1-h incubation with Goat anti-Rabbit IgG H & L (HRP) (1:2,000, Abcam, Cambridge, UK) secondary antibody. The membranes were added with the enhanced chemiluminescence (ECL) kit (#P1000, Applygen, Beijing, China), and Tanon-3500 Image Analyzer (Tanon, Shanghai, China) was employed to visualize the protein bands.

### Statistical analysis

All the data were exhibited as mean ± standard deviation (SD). Each experiment was repeated thrice. GraphPad 7.0 software was applied for statistical analysis. A one-way ANOVA test was employed to compare group difference. *p* < 0.05 indicates marked difference.

## Results

### DHM inhibits cell viability, migration, invasion, and EMT, as well as promoting apoptosis of MM cells

**Figure 1:**
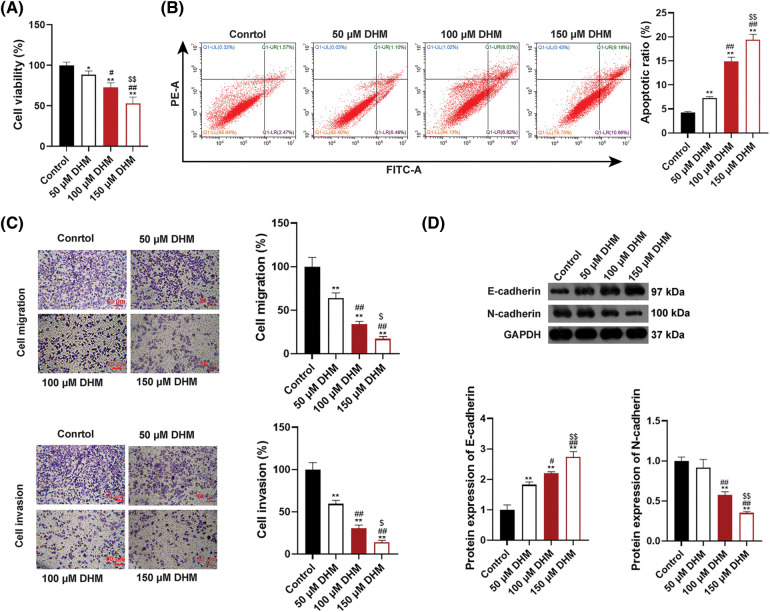
Effects of DHM on the malignant characteristics of U266 cells. (A) Detection of viability in U266 cells by CCK-8. (B) Detection of apoptosis in U266 cells by flow cytometry. (C) Detection of migration and invasion in U266 cells by trans-well. Scale bar = 50 μm. (D) Detection of the protein levels of E-cadherin and N-cadherin in U266 cells by western blotting. *p < 0.05 and **p < 0.01 *vs*. Control group; ^#^*p* < 0.05 and ^##^*p* < 0.01 *vs*. 50 μM DHM group; ^$^*p* < 0.05 and ^$$^*p* < 0.01 *vs*. 100 μM DHM group. DHM, dihydromyricetin; MM, multiple myeloma.

To verify the effects of DHM on MM, the viability of MM cell line U266 was detected. Fig. 1A demonstrated that DHM restrained the viability of U266 cells in a dose-dependent way (*p* < 0.05). Also, Fig. 1B presented that the apoptosis ratio of U266 cells was significantly enhanced after DHM administration in a dose-dependent way (*p* < 0.01). Additionally, as displayed in Fig. 1C, the number of migrated and invaded U266 cells was remarkably down-regulated by DHM treatment in a dose-dependent way (*p* < 0.05, Fig. 1C). The cell-biological program, EMT, has implications for tumor development and progression [34,35]. E-cadherin is an epithelial marker decreased during EMT, whereas N-cadherin is a mesenchymal marker increased during EMT [36]. To figure out the role of DHM on EMT in MM cells, the protein expression levels of EMT indicators, E-cadherin and N-cadherin, were detected in U266 and RPMI-8226 cells. According to Fig. 1D, DHM dose-dependently elvated E-cadherin level and reduced the N-cadherin level in U266 cells (*p* < 0.05). Besides, DHM exerted similar effects on elevating E-cadherin level and reducing N-cadherin level in RPMI-8226 cells (*p* < 0.05, Suppl. Fig. S1).

### STAT1/RIG-I pathway is blocked in MM plasma and cells

**Figure 2:**
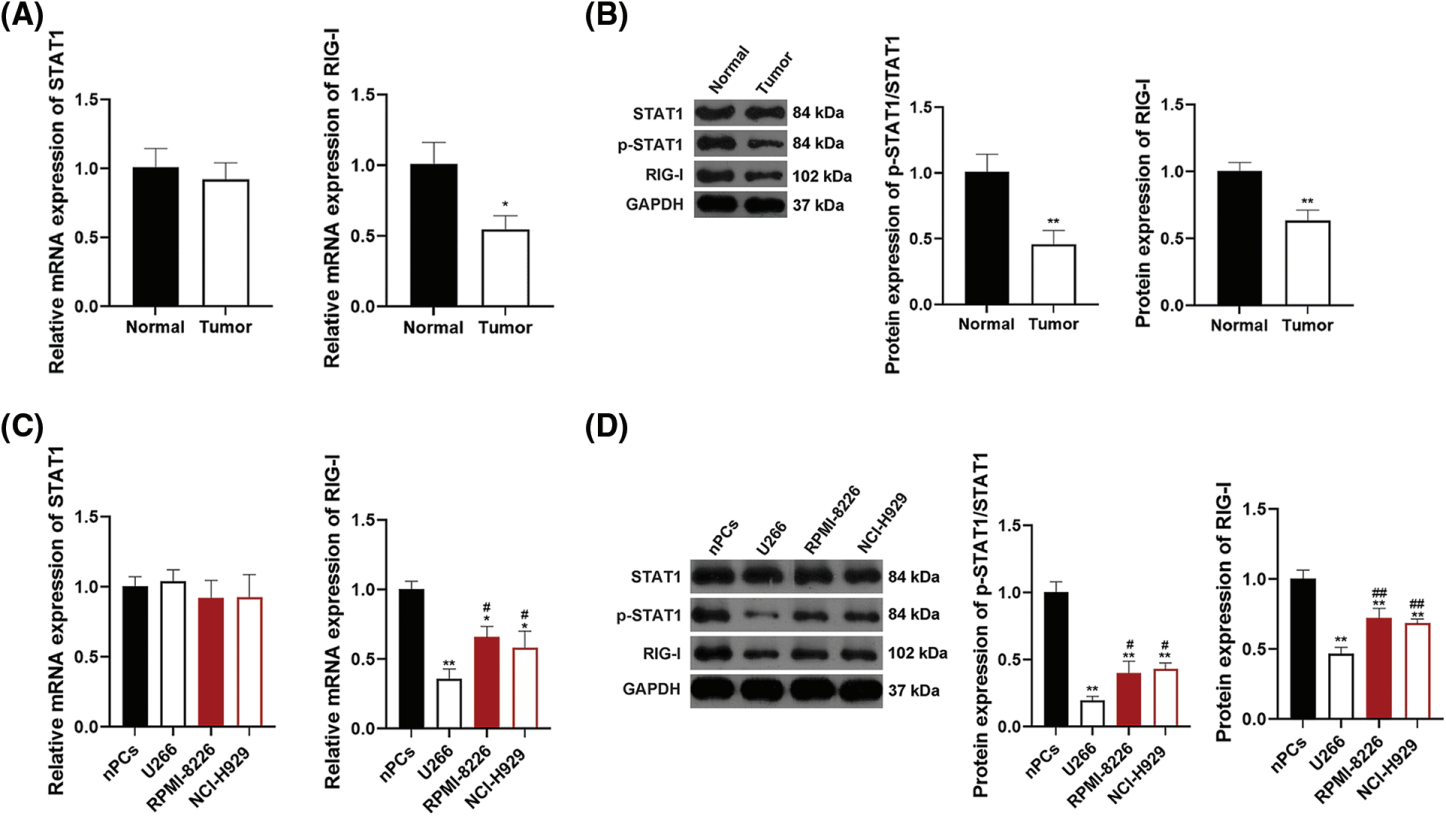
The STAT1/RIG-I pathway is blocked in MM. (A) Detection of mRNA levels of STAT1 and RIG-I by qRT-PCR. (B) Detection of protein levels of p-STAT1/STAT1 and RIG-I in MM tumor and normal plasma samples by western blotting. *p < 0.05 and **p < 0.01 *vs*. Normal group. (C) Detection of mRNA levels of STAT1 and RIG-I in MM cell lines (U266, RPMI-8226, and NCI-H929) and nPCs by qRT-PCR. (D) Detection of protein levels of p-STAT1/STAT1 and RIG-I in MM cell lines (U266, RPMI-8226, and NCI-H929) and nPCs by western blotting. *p < 0.05 and **p < 0.01 *vs*. nPCs group; ^#^*p* < 0.05 and ^##^*p* < 0.01 *vs*. U266 group. MM, multiple myeloma; nPCs, normal plasma cells.

Research has revealed that the STAT1/RIG-I axis plays a significant part in anti-tumor processes [30,37]. To determine whether there is a connection between the STAT1/RIG-I pathway and MM, the mRNA expression of STAT1 and RIG-I and protein expression of STAT1, p-STAT1, and RIG-I were assessed in human MM and normal plasma samples. As shown in Fig. 2A, RIG-I mRNA expressionin tumor plasma was notably lower than those in normal plasma (*p* < 0.05). Protein expression levels of p-STAT1/STAT1 and RIG-I were also down-regulated in tumor plasma in comparison to those in normal plasma (*p* < 0.01, Fig. 2B). To further ascertain the mechanism of the STAT1/RIG-I pathway in MM, the related mRNA and protein expression were assessed in MM cell lines, U266, RPMI-8226, NCI-H929, as well as nPCs. Fig. 2C revealed that the mRNA expression levels of RIG-I in MM cell lines (U266, RPMI-8226, NCI-H929) were notably lower than those in nPCs, especially in U266 cells (*p* < 0.05). Protein expression levels of p-STAT1/STAT1 and RIG-I in MM cells were also observably lower than those in nPCs, particularly in U266 cells (*p* < 0.05, Fig. 2D). Accordingly, U266 cells were selected for subsequent assays.

### DHM suppresses the viability, migration, invasion, and EMT, and enhances the apoptosis of MM cells via promoting the STAT1/RIG-I axis

**Figure 3:**
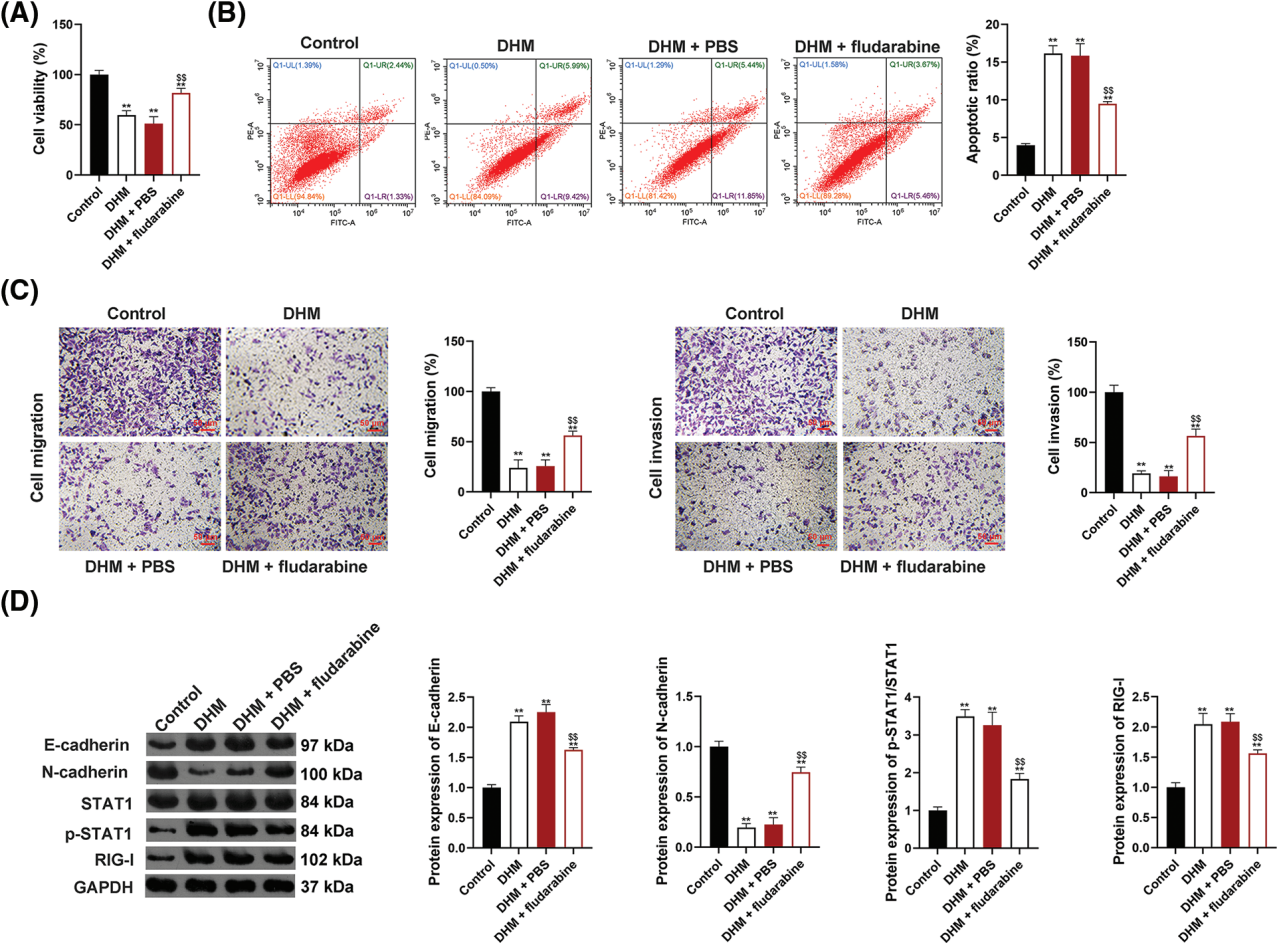
Effects of DHM on the malignant features of MM cells by up-regulating the STAT1/RIG-I pathway. (A) Detection of viability of U266 cells by CCK-8. (B) Detection of apoptosis of U266 cells by flow cytometry. (C) Detection of the migration and invasion of U266 cells by trans-well. Scale bar = 50 μm. (D) Detection of protein levels of E-cadherin, N-cadherin, p-STAT1/STAT1, and RIG-I by western blotting. **p < 0.01 *vs*. Control group; ^$$^*p* < 0.01 *vs*. DHM+PBS group. DHM, dihydromyricetin; MM, multiple myeloma.

To determine whether DHM restrains MM via targeting the STAT1/RIG-I axis, the viability of U266 cells was detected after administration with DHM and/or fludarabine (a STAT1 inhibitor). As shown in Fig. 3A, fludarabine abolished the suppressive functions of DHM in U266 cell viability (*p* < 0.01). Also, fludarabine undid the promotive functions of DHM in U266 cell apoptosis (*p* < 0.01, Fig. 3B). Besides, Fig. 3C exhibits that fludarabine eliminated the suppressive role of DHM in U266 cell migration and invasion (*p* < 0.01). The STAT1/RIG-I pathway-related and EMT-related proteins were assessed in U226 cells after treatment with DHM and/or fludarabine to further verify the mechanism of DHM affecting EMT in MM cells. Fig. 3D showed that fludarabine undid the functions of DHM in E-cadherin up-regulation and N-cadherin down-regulation, with p-STAT1/STAT1 and RIG-I levels falling (*p* < 0.01).

### DHM restrains MM tumor growth and EMT in vivo by facilitating the STAT1/RIG-I pathway

**Figure 4:**
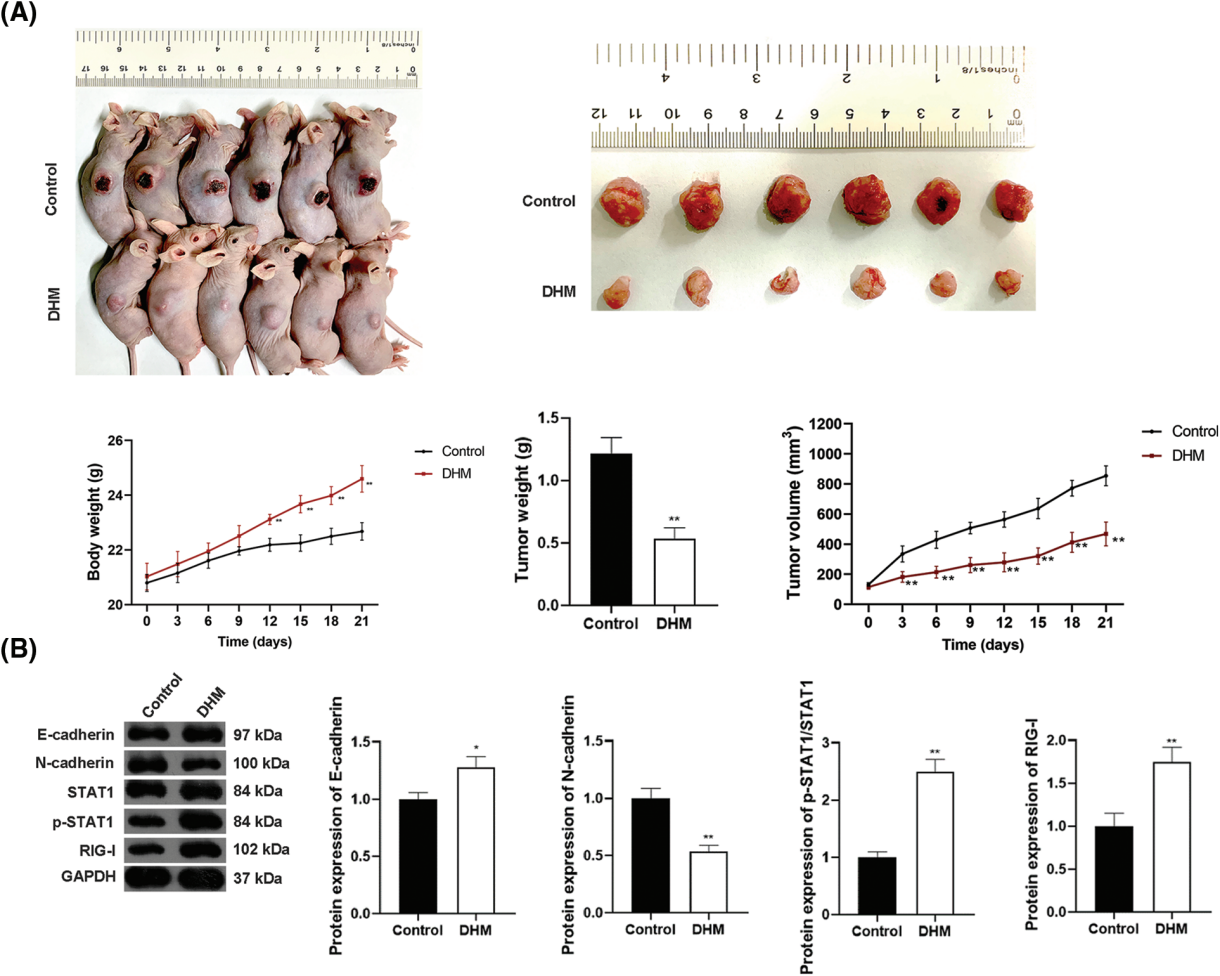
Effects of DHM on MM tumor growth and EMT in xenograft mice by promoting the STAT1/RIG-I pathway. (A) Detection of the body weights of xenograft mice, as well as MM tumor weights and volumes in xenograft mice. (B) Detection of protein levels of E-cadherin, N-cadherin, p-STAT1/STAT1, and RIG-I in xenograft mice by western blotting. *p < 0.05 and **p < 0.01 *vs*. Control group. DHM, dihydromyricetin; MM, multiple myeloma; EMT, epithelial-mesenchymal transition.

To validate the effects of DHM on inhibiting MM tumor growth *in vivo*, the xenograft mouse model was built and treated with DHM. Fig. 4A showed that the body weights of xenograft mice notably ascended after DHM treatment, and the weights and volumes of tumors in the xenograft mice markedly descended (*p* < 0.01). Also, to determine whether DHM exerts inhibitory effects on EMT *in vivo*, E-cadherin and N-cadherin expression was assessed in the xenograft tumor tissues. After DHM administration, E-cadherin protein level observably rose, whereas N-cadherin protein level dropped (*p* < 0.05, Fig. 4B). The STAT1/RIG-I pathway was also analyzed in the xenograft tumor tissues to validate the mechanism by which DHM inhibits tumor growth and EMT *in vivo*. As shown in Fig. 4B, DHM markedly elevated the protein levels of p-STAT1/STAT1 and RIG-I (*p* < 0.01).

**Figure 5:**
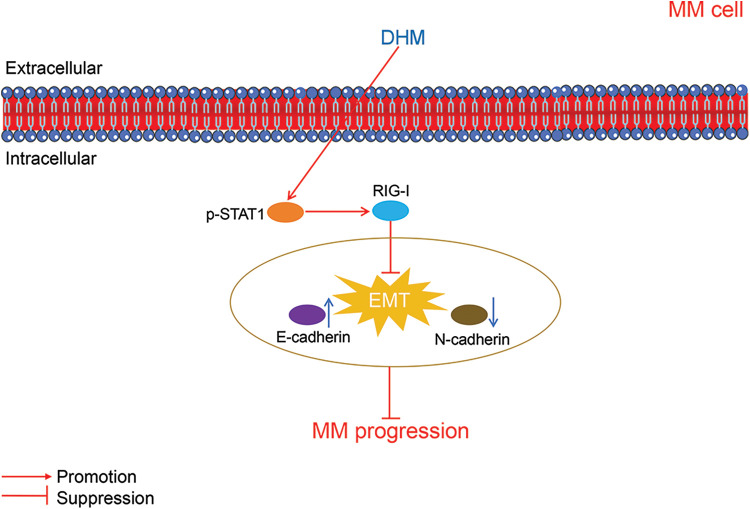
The schematic schematic diagram showing the mechanisms of action of DHM against MM. MM, multiple myeloma; DHM, dihydromyricetin; EMT, epithelial-mesenchymal transition.

The mechanisms through which DHM suppressed MM progression were displayed in Fig. 5.

## Discussion

MM is a plasma cell hematologic malignancy of bone marrow, which commonly triggers end-organ injury, including anemia, renal impairment, lytic bony impairment, as well as hypercalcemia [38]. Despite notable advances in patient outcomes accompanied by myeloma-targeted and immunomodulatory agents, MM remains largely incurable. Therefore, the development of novel and practical drug therapy is urgently required. In recent years, TCM has been widely applied in cancer treatment in China. TCM has been proven to assist in suppressing tumor growth and enhancing the survival rates of cancer patients [39,40]. Herein, the Chinese herbal ingredient, DHM, exhibited anti-tumor effects on MM by inhibiting cell viability, migration and invasion, EMT, and tumor growth, as well as promoting cell apoptosis. Additionally, the STAT1/RIG-I pathway was implicated in the anti-MM mechanism of DHM.

DHM, a natural component isolated from *Ampelopsis grossedentata*, exhibits various biological functions in some cancers. For example, DHM has presented an inhibitory effect on cell viability in cholangiocarcinoma [19]. DHM can inhibit cell proliferation, motivate apoptosis, and regulate redox balance in liver cancer cells [12]. Furthermore, DHM has been indicated to suppress cell migration and invasion in osteosarcoma cell lines [41]. Cell migration and invasion are crucial stages during tumor progression [42]. EMT is a dynamic cellular process that has crucial implications for embryogenesis, malignant progression, and disorders, like cancer invasion and metastasis. During EMT, polarized epithelial cells undergo a loss of their adhesion characteristics and adopt phenotypes resembling mesenchymal cells [43,44]. Besides, cancer cells enhance their capacity for migration, invasion, and metastasis through the mechanism of EMT with a series of gene expression changes. For instance, E-cadherin expression is enhanced, whereas N-cadherin expression is suppressed during EMT [19]. Increasingly studies reveal that DHM has the potential to mitigate some malignancies, including cholangiocarcinoma [19] and esophageal squamous cell carcinoma [45] by restraining EMT. Our data showed that DHM dose-dependently weakened the viability and enhanced apoptosis of MM cells. Meanwhile, MM cell migration and invasion were notably restrained by DHM in a dose-dependent way. DHM also observably enhanced E-cadherin expression and lowered N-cadherin expression. Taken together, these data suggest that DHM can suppress the malignant progression of MM cells.

Previous research showed that the STAT1/RIG-I axis has significant implications for tumor development and pathogenesis [29,30,46,47]. For example, RIG-I acts as a tumor inhibitor via enhancing STAT1 activation in hepatocellular carcinoma and acute myeloid leukemia [29]. According to our data, p-STAT1/STAT1 and RIG-I levels in MM tumor plasma and cell lines were observably lowered relative to those in normal plasma and cells. This indicates the suppression of the STAT1/RIG-I pathway in MM.

Evidence has demonstrated that STAT1 can interact with RIG-I, which is vital for the regulation of EMT [29]. For example, targeting STAT1 can promote EMT in glioma [48] and lung adenocarcinoma cells [49]. Hence, we hypothesized that the STAT1/RIG-I axis might be a mechanical target of DHM against EMT in MM cells. Our results showed that the STAT1 inhibitor fludarabine abolished the effects of DHM restraining MM cell viability, migration, invasion, and EMT, and accelerated apoptosis. This implies that DHM suppresses the malignant progression of MM cells by stimulating the STAT1/RIG-I axis.

Evidence indicates that DHM can directly restrain tumor growth *in vivo* [19,50,51]. For instance, DHM can inhibit tumor growth of nasopharyngeal cancer [50] and hepatocellular carcinoma [51] in xenograft mice. Our results demonstrated that the weights of xenograft mice were up-regulated after DHM treatment, while the weights and volumes of MM tumors were notably down-regulated. Meanwhile, DHM notably elevated E-cadherin expression and reduced N-cadherin expression in xenograft mice. Besides, p-STAT1/STAT1 and RIG-I protein levels in xenograft mice markedly dropped after DHM administration. Collectively, these results suggest that DHM can inhibit MM tumor growth and EMT *in vivo* via STAT1/RIG-I activation.

However, this work has certain limitations. The pathway by which DHM prevented MM progression was verified at the *in vitro* level without *in vivo* verification. Besides, the anti-MM effects of DHM remain to be clinically validated, and related mechanisms should be. In our future research, in-depth experiments will be carried out to further explore the anti-MM mechanisms of DHM.

## Conclusion

In summary, this study revealed that DHM exerts therapeutic effects on MM by inhibiting viability, migration, invasion, EMT, and tumor growth, as well as enhancing apoptosis via STAT1/RIG-I activation. The findings of this study provide a direction for follow-up clinical research on the efficacy of DHM. Additionally, further research into the pharmacokinetics and pharmacodynamics of DHM can be conducted to establish the optimal treatment regimens. On the other hand, since this study revealed that the activation of the STAT1/RIG-I pathway by DHM is beneficial for the treatment of MM, future research can focus on this pathway to develop targeted agents for enhanced therapeutic effects.

## Supplementary Materials

**Figure S1:**
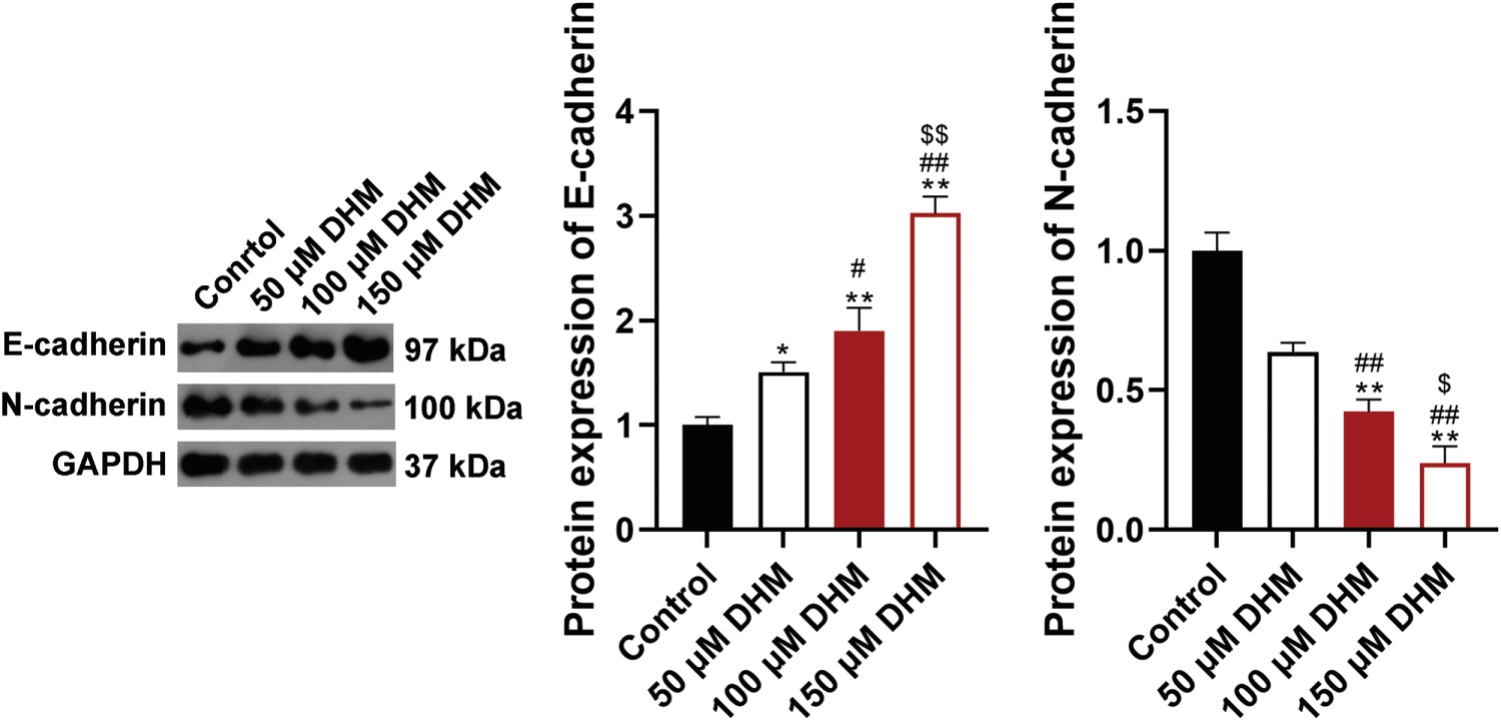
Effects of DHM on the EMT of RPMI-8226 cells. Detection of the protein levels of E-cadherin and N-cadherin in RPMI8226 cells by western blotting. *p < 0.05 and **p < 0.01 *vs*. Control group; ^#^*p* < 0.05 and ^##^*p* < 0.01 *vs*. 50 μM DHM group; ^$^*p* < 0.05 and ^$$^*p* < 0.01 *vs*. 100 μM DHM group. DHM, dihydromyricetin; EMT, epithelial-mesenchymal transition.

## Data Availability

The datasets generated and analyzed during the current study are available from the corresponding author on reasonable request.

## Abbreviations

MM: Multiple myeloma
DHM: Dihydromyricetin
qRT-PCR: Quantitative real-time polymerase chain reaction
EMT: Epithelial-mesenchymal transition
MGUS: Monoclonal gammopathy of uncertain significance
TCM: Traditional Chinese medicine
RIG-I: Retinoic acid inducible gene I
STAT1: Signal transducer and activator of transcription 1
nPC: Normal plasma cell
RPMI: Roswell Park Memorial Institute
FBS: Fetal bovine serum
PBS: Phosphate buffer saline
CCK-8: Cell counting kit-8
OD: Optical density
FITC: Fluorescein isothiocyanate
PI: Propidium iodide
RIPA: Radioimmunoprecipitation assay
BCA: Bicinchoninic acid
PVDF: Polyvinylidene difluoride
TBST: Tris-buffered saline with Tween-20
ECL: Enhanced chemiluminescence
SD: Standard deviation
